# Association of Septic Shock with Mortality in Hospitalized COVID-19 Patients in Wuhan, China

**DOI:** 10.1155/2022/3178283

**Published:** 2022-04-23

**Authors:** Shaoqiu Chen, Zitong Gao, Ling Hu, Yi Zuo, Yuanyuan Fu, Meilin Wei, Emory Zitello, Gang Huang, Youping Deng

**Affiliations:** ^1^Department of Quantitative Health Sciences, John A. Burns School of Medicine, University of Hawaii at Manoa, Honolulu, HI 96813, USA; ^2^Molecular Biosciences and Bioengineering Program, College of Tropical Agriculture and Human Resources, University of Hawaii at Manoa, Honolulu, HI 96822, USA; ^3^Tianyou Hospital, Wuhan University of Science and Technology, Wuhan, Hubei 430065, China; ^4^Department of Metabolism and Endocrinology, The Second Affiliated Hospital of Nanchang University, Jiangxi 330006, Nanchang, China; ^5^Shanghai Key Laboratory for Molecular Imaging, Shanghai University of Medicine and Health Sciences, Shanghai 201318, China

## Abstract

**Purpose:**

Septic shock is a severe complication of COVID-19 patients. We aim to identify risk factors associated with septic shock and mortality among COVID-19 patients.

**Methods:**

A total of 212 COVID-19 confirmed patients in Wuhan were included in this retrospective study. Clinical outcomes were designated as nonseptic shock and septic shock. Log-rank test was conducted to determine any association with clinical progression. A prediction model was established using random forest.

**Results:**

The mortality of septic shock and nonshock patients with COVID-19 was 96.7% (29/30) and 3.8% (7/182). Patients taking hypnotics had a much lower chance to develop septic shock (HR = 0.096, *p*=0.0014). By univariate logistic regression analysis, 40 risk factors were significantly associated with septic shock. Based on multiple regression analysis, eight risk factors were shown to be independent risk factors and these factors were then selected to build a model to predict septic shock with AUC = 0.956. These eight factors included disease severity (HR = 15, *p* < 0.001), age > 65 years (HR = 2.6, *p*=0.012), temperature > 39.1°C (HR = 2.9, *p*=0.047), white blood cell count > 10 × 10⁹ (HR = 6.9, *p* < 0.001), neutrophil count > 75 × 10⁹ (HR = 2.4, *p*=0.022), creatine kinase > 5 U/L (HR = 1.8, *p*=0.042), glucose > 6.1 mmol/L (HR = 7, *p* < 0.001), and lactate > 2 mmol/L (HR = 22, *p* < 0.001).

**Conclusions:**

We found 40 risk factors were significantly associated with septic shock. The model contained eight independent factors that can accurately predict septic shock. The administration of hypnotics could potentially reduce the incidence of septic shock in COVID-19 patients.

## 1. Introduction

By June 07, more than seven million people developed cases of coronavirus disease 2019 (COVID-19) and over 400,000 people died worldwide. Several published articles have proven that the current standard of treatment is often ineffective in critical situations and have identified several risk factors associated with bad outcomes for diagnosed patients [1, 2].

Sepsis and septic shock are systemic inflammatory conditions associated with various infectious diseases such as pneumonia [3], influenza [4], and urinary tract infections [5]. Sepsis is a common and frequent clinical condition that is associated with substantial mortality. Some studies showed that sepsis and septic shock account for nearly 30–50% of deaths in intensive care unit (ICU) [6–8]. The cytokine‐mediated hyper‐inflammatory phase and a subsequent immunosuppressive phase are features of the immune response during sepsis [9]. Immunodeficiency highly impacts on the progression of sepsis to septic shock. A COVID-19 study of 150 patients from China indicated that 16% of patients who died had secondary infection, while 1% of discharged patients experienced secondary infection [10]. Another study also found that 6% of patients experienced septic shock during hospitalization [11].

However, there is no original research published that analyzes the causes of septic shock or how septic shock affects mortality, and no risk factors associated with shock have been described among COVID-19 patients. In this retrospective study, we analyzed the records of 212 COVID-19-infected patients admitted to Wuhan Tianyou Hospital to identify risk factors related to septic shock and mortality.

## 2. Methods

### 2.1. Study Design

The institution of Tianyou Hospital, an affiliate of the Wuhan University of Science and Technology, approved the retrospective study and ethical design. Oral consent was obtained from all patients. This is a randomized retrospective study. A total of 212 reverse transcription-polymerase chain reaction (RT-PCR)-confirmed patients with COVID-19 hospitalized between January 14 and March 1, 2020, were included. The final follow-up date was March 16, 2020. Initially, data for 402 patients were gathered; after excluding PCR-negative cases and patients with incomplete medical records, a final total of 212 patients were included in our study.

### 2.2. Data Collection and Participants

We obtained data concerning the clinical symptoms, laboratory findings, and treatment of patients from electronic medical records. Computed tomography (CT) and laboratory tests were offered according to clinical needs. All medical records were assessed by specifically trained physicians. The detailed methods of laboratory testing, medication, and clinical complications are provided in the appendix.

### 2.3. Diagnosis and Case Definition

Laboratory confirmation of COVID-19 by RT-PCR was used as a diagnostic standard. The RT-PCR assays were performed according to previous reports [12]. We defined septic shock and categorized the patients into three groups: mild, severe, and critical in accordance with the WHO interim guidance and guidelines of COVID-19 diagnosis [13] and treatment trial (5th edition), by the National Health Commission of the People's Republic of China [14].

### 2.4. Statistics Analysis

For categorical variables, Fisher's exact test or the chi-squared test was used in the analyses presented here. Student's *t*-test or Mann–Whitney U-test was used for tests of continuous variables. Continuous variables were formulated as medians and interquartile ranges (IQR). The Kaplan–Meier method and the log-rank test were performed for cases of septic shock associated with survival status. The prediction model was formulated using a random forest algorithm. Hazard ratios (HRs) and 95% confidence intervals (CIs) were computed using multivariate logistic regression models. All analyses were executed with the R software (version 4.0.0).

## 3. Results

### 3.1. Clinical Outcomes

**Figure 1:**
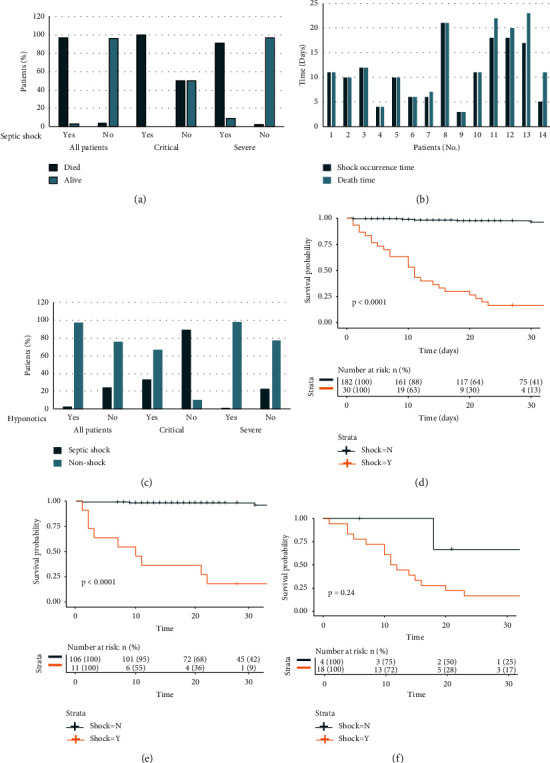
Clinical outcomes and survival analysis. (a) Clinical outcomes of COVID-19 patients with septic shock in all patients, in severely ill patients, and in the critically ill group. The survival rates are calculated by the survival of individual patients divided by the entire number of patents in each group. (b) Time to onset of septic shock and time to death after hospitalization, 9 patients had the same duration of septic shock following onset and time to death; the longest interval was 6 days in patient number 14 (some patients had no clear onset of septic shock); No, Number. (c) The septic shock occurrence rates of patients who took hypnotics shown in critical and severe categories. (d) Kaplan–Meier curve of COVID-19 patients with septic shock including all patients; patients in the nonseptic shock had significantly better survival rates (*p* < 0.0001). (e) Kaplan–Meier curve of COVID-19 patients without septic shock had significantly better survival rates in the severe group (*p* < 0.05). (f) Kaplan–Meier curve of COVID-19 patients with septic shock had a trend toward worse survival rates in the critical group (*p* < 0.24).

Of the 212 RT-PCR-confirmed COVID-19 patients, 30 (14.2%) cases progressed to septic shock and 182 (85.8%) did not (Table 1). Septic shock patients were categorized into three groups based on severity, including 1 mild (3.4%), 11 severe (36.6%), and 18 critical (60%) cases, respectively. By the final follow-up date of March 16, 2020, thirty-six patients had died, and 176 patients had been discharged. The overall cure at the time of discharge was 83%. Of the 36 deceased individuals, 96.7% were patients with septic shock. The death rate for patients who developed septic shock was 96.7%. By contrast, the survival rate of patients without septic shock was 96.2% (Figure 1(a)).

The mortality rates of patients presenting with septic shock in the critical and severe groups were 100% and 90.9%, respectively. Of the 182 nonseptic shock patients, 50% and 9.1% died in the critical and severe groups (Figure 1(a)). We also found that the interval of occurrence time of septic shock and time until death was particularly close among those patients who experienced septic shock. We present the survival time of 14 critical patients with septic shock in Figure 1(b); 9 patients succumbed on the day of occurrence of septic shock. The longest interval among these was 6 days, which occurred in 14 patient cases.

Table 1 shows that the median age of septic shock patients (73.5, IQR 59–84) was significantly higher than that of nonseptic shock patients (61, IQR 51.25–68). Retired individuals were overrepresented within the septic shock group (24/30, 80%). However, there was no gender difference. The number of patients with a smoking history, BMI > 30, temperature ≥ 39.01 (fever and cough), diabetes, cardiovascular and cerebrovascular diseases, and respiratory system disease were significantly greater in the septic shock group (*p* < 0.05). Notably, the occurrence rate of septic shock was significantly different (*p* < 0.01) between those who took hypnotics (6.7%) and those who did not (93.3%). The septic shock occurrence rates of patients who took hypnotics in the critical and severe categories were 33.3% and 1.7%, respectively. Among all patients, 89.4% and 22.7% of patients not receiving hypnotics experienced septic shock in the critical and severe groups (Figure 1(c)). The survival rates of patients who took hypnotics in critical and severe cases were 66.7% and 98.2%. 100% and 27.3% of patients not receiving hypnotics died in the critical and severe groups (Supplementary Figure S1).

### 3.2. Radiologic and Laboratory Abnormalities

Table 2 is a compilation of the results of computed tomography (CT) and laboratory findings (the full table is prepared as Supplementary Table S1). Based on CT images, the so-called “crazy paving sign” (6/30, 20%), bilateral pulmonary multiple consolidation, and intralobular interstitial thickening (6/30, 20%) were more common among patients with septic shock. Nonetheless, the combination of patchy ground-glass opacity and pulmonary consolidation was more frequent in the nonseptic group. To illustrate this point, chest computer tomography (CT) scans from one COVID-19 patient with septic shock are included as Supplementary Figure S2. From the initial hospitalization to the fifth day, the progression was clearly showed under the right lung pleura with the appearance of a patchy membrane glass shadow. The lesions of both lungs increased with time, and the lesions of the right lung progressed more than seen in previous radiographs.

Significantly different laboratory results between septic shock and nonseptic groups were seen in white blood cell counts, neutrophil counts, lymphocyte counts, platelet counts, activated partial thromboplastin time, creatine kinase, alanine amino transferase (ALT), aspartate aminotransferase (AST), blood urea nitrogen (BUN), creatinine, and glucose. Glucose in excess of 6.1 mmol/L was present in 80.8% of patients who experienced septic shock but only in 35.2% of patients with no septic shock outcome (*p* < 0.01).

### 3.3. Treatments and Complications

Table 3 shows the results of treatment and complications. Arbidol (17/30, 56.7%) and Kaletra® (lopinavir/ritonavir) (11/30, 36.7%) were the two most regularly used antiviral medications in the septic shock group. For oxygen support, nasal cannula was the most commonly used treatment; only 2 patients received no support *via* nasal cannula (210/212, 99.1%). Patients who required noninvasive ventilation (27/30, 90%) and invasive mechanical (17/30, 56.7%) were distributed more often in the septic shock group.

In the septic group, acute cardiac injury (24/30, 80%), arrhythmia (18/30, 60%), acute respiratory distress syndrome (28/30, 93.3%), acute kidney injury (9/30, 30%), acute respiratory injury (29/30, 96.7%), and secondary infection (8/30, 26.7%) occurred significantly more often than in the septic shock-free group.

### 3.4. Survival Analysis, Hazard Rations, and Prediction Model

**Figure 2:**
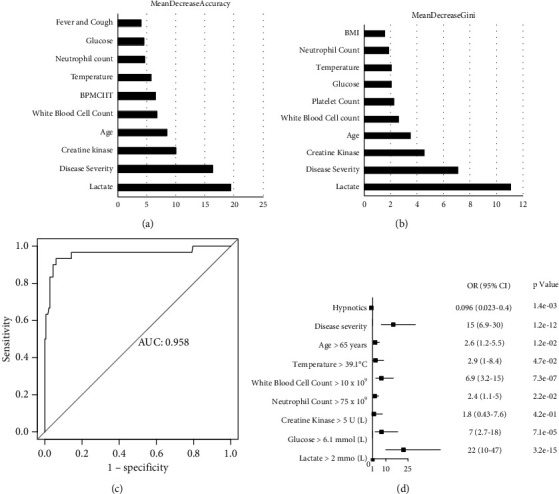
Prediction model and multivariate regression. (a) Mean Decrease Accuracy shows the relative degree to which a factor improves the accuracy of the forest in classification prediction. (b) Mean Decrease Gini assigns a weight of importance to each parameter, which improves accuracy of the prediction. (c) A receiver operating characteristic curve (ROC curve) based on random forest algorithm predicts COVID-19 patients develop septic shock. (d) Independent significant factors for septic shock in hazard ratio analysis based on a multivariate logistic regression model show that hypnotics (HR = 0.096, *p*=0.0014) were the only protecting factor.

All patients without septic shock had significantly better survival rates (*p* < 0.0001, Figure 1(d)); a similar difference was also observed when comparing severe (*p* < 0.0001, Figure 1(e)) and critical groups (*p* < 0.05, Figure 1(f)). Using the Mean Decrease Accuracy and Mean Decrease Gini packages based on Random Forest (Figures 2(a) and 2(b)), 8 variables were selected based upon their association with mortality and performed well in the model, including age, severity status, blood glucose level, white blood cell count, neutrophil count, temperature, creatine kinase, and lactate.  Figure 2(c) shows the performance of the prediction model with an area under the ROC curve (AUC) of 0.958.  Figure 2(d) showed that a total of nine variables were determined as independent risk factors based on a multivariate logistic regression model. The 9 factors included 8 factors in the prediction model plus the administration of hypnotics. The results showed that hypnotics (HR = 0.096, *p*=0.0014), disease severity (HR = 15, *p* < 0.001), age > 65 years (HR = 2.6, *p*=0.012), temperature > 39.1°C (HR = 2.9, *p*=0.047), white blood cell count (WBC) > 10 × 10⁹ (HR = 6.9, *p* < 0.001), neutrophil count > 75 × 10⁹ (HR = 2.4, *p*=0.022), creatine kinase > 5 U/L (HR = 1.8, *p*=0.042), glucose greater than 6.1 mmol/L (HR = 7, *p* < 0.001), and lactate above 2 mmol/L (HR = 22, *p* < 0.001) were significantly correlated with patient outcomes. To understand the clinical change in the development of septic shock, 5 completed dynamic laboratory features selected by the prediction model are presented in Figure 2(d). During the admission day to day 14, white blood cell count, neutrophil count, blood urea nitrogen, and glucose were significantly increased in the septic shock group. Predictably, lymphocyte count and platelet count significantly decreased (Supplementary Figure S3).

## 4. Discussion

We found COVID-19 patients with septic shock complications had a much higher death rate than those without septic shock. This implies that early prediction and/or detection of septic shock can provide crucial guidance for the treatment of COVID-19 patients and prevention of patient death. We used the clinical factors and laboratory test indicators at the time of the patient's admission to construct a prediction model. After optimizing the area under the receiver operating characteristic curve (AUC) of the random forest (RF), we obtained a considerable AUC area (0.958), suggesting the model's utility to predict septic shock. The eight parameters determined by the model at their respective individual weights can be validated in future cases to predict, analyze, and treat septic shock. Our purpose is to find best risk factors for predicting septic shock and build a model. Other risk factors may also be related to septic shock, but we find the best combination that fits the model through the machine learning algorithm.

In this study, we found septic shock mainly occurred among the critically ill patients (81.8%), and it was in accord with the finding that septic shock was associated with advanced age of patients (73.5 vs 61 years of age). It has been proven by several studies that younger patients have higher survival rates, less complications, and less severe disease, while in patients of higher mean age, severely and critically ill disease [1, 15] are typical. Others have shown that COVID-19 infection is more likely to be associated with underlying diseases such as hypertension, diabetes, and malignancy. It was shown in the analysis of Hu et al. that smoking history, BMI, and diabetes were risk factors for poorer clinical outcome; these investigators also validated previous findings that BMI over 31, diabetes, and smoking history were associated with patients who experienced septic shock [2].

Blood lactate level is the most important variable in our model and other studies of septic shock [16, 17]. Although many people have recognized the role of lactate in septic shock, the origin of lactate has received less mention. With prolonged tissue hypoxia, anaerobic metabolism converts glucose into lactate. Laboratory testing showed that blood glucose levels increased significantly more during the 14-day observation period in the septic shock group than in the nonseptic shock group. Hyperglycemia is a frequent and important metabolic derangement that accompanies septic shock [18]. Although the finding of increased blood glucose may be caused by septic shock, it may also be caused by drugs; both lopinavir and glucocorticoids are known to cause an increase in blood glucose [19, 20]. Notably, 90% of patients with septic shock received glucocorticoids, significantly higher than in the nonseptic shock group (*p* < 0.05). Elevated blood glucose can increase the generation of oxygen free radicals that cause inflammatory stress by activating redox-sensitive pro-inflammatory transcription factors [21, 22] and the pro-inflammatory cytokines. Excessive production of pro-inflammatory cytokines that perturb normal regulation of the immune response can induce pathological inflammatory responses, such as capillary leakage, tissue injury, and lethal organ failure [23]. Moreover, septic shock can result from the imbalance of inflammation, bacterial or viral infection, and a complicated interaction with the host immune system, which occasionally triggers an intense inflammatory response or excessive inflammation, sometimes referred to as “cytokine storm” [10, 24, 25].

For patients with septic shock caused by COVID-19, the onset of shock and time to death are very closely related chronologically, and many patients appear to have signs of septic shock soon before death (Figure 1(b)).

However, for patients prescribed hypnotics, dexzopiclone at a dose of 1.0 mg per day was significantly correlated with more favorable clinical outcomes. Only 2 of 68 (6.7%) patients to whom the hypnotics were administered progressed to exhibit signs consistent with clinical septic shock. We performed the multivariate analysis (Figure 2(d)) to identify other factors. The hazard ratio showed that hypnotics were the only significant independent protecting factor (*p*=0.0014) and that hypnotics could significantly improve COVID-19 patient outcomes [2]. It indicated that hypnotics could assist patients in staying calm throughout 2 to 3 weeks of hospitalization, which contributed to the improved survival and recovery rate (Figure 1(c)). Previous studies showed that upregulation of integrin activation through improved rest can be an underlying immune-supportive mechanism and can potentially enhance effective T-cell responses [26]. Moreover, dexzopiclone may enhance gamma-aminobutyric acid (GABA) signaling by interacting with GABA_A_ to promote autophagy activation. Through more in-depth research, dexzopiclone may not only help patients fight infection by improving immunity but also may help patients resist septic shock by regulating metabolism [27]. Abnormal sleep can cause abnormal glucose metabolism to exacerbate existing endocrine conditions and adequate sleep can regulate glucose homeostasis, thereby reducing the occurrence of septic shock [28].

In this study, acute cardiac injury and acute respiratory distress syndrome (ARDS) were two significant complications among septic shock and nonseptic shock patient groups. It has been proven that a majority of patients with sepsis experienced myocardial injury, an independent factor associated with early mortality [29]. One autopsy report result showed that typical ARDS syndrome occurred in the lungs bilaterally and manifested with the overactivation of T cells, by increasing the number of Th17 cells and through high cytotoxicity of CD8+ T cells, which contributed to the severe immune injury in this patient [30].

Septic shock is one of the most severe complications related to secondary infection and higher mortality of COVID-19 patients. Currently, no effective standard treatment can be applied for COVID-19, and targeted immune-enhancing therapy may be a valid approach in selected patients with sepsis. However, the specific immunological mechanism related to the occurrence of cytokine storm has not been fully explained. Although some clinical trials using anticytokine storm drugs have been conducted, there have still been many adverse reactions in these patient trials, preventing their application in a wide range of clinical situations. Through retrospective studies, we found that septic shock is an important risk factor for death of the COVID-19, which indicates that septic shock may have a causal relationship with death, which requires further clinical experiments to verify.

## Figures and Tables

**Table 1:** Demographics and clinical outcomes in patients with COVID-19.

	All patients	Clinical outcomes		*p* value
		Septic shock	Nonseptic shock	
	212	30	182	
Characteristic				
Age (years)—median (IQR)	62 (53–70)	73.5 (59–84)	61 (51.25–68)	<0.001
Age (years)—No. (%)				0.019
20–40	17/212 (8)	2/30 (6.7)	15/182 (8.2)	1.000
41–65	114/212 (53.8)	9/30 (30)	105/182 (57.7)	0.005
≥65	88/212 (41.5)	19/30 (63.3)	69/182 (37.9)	0.009
Sex—No. (%)				0.914
Male	115/212 (54.2)	16/30 (53.3)	99/182 (54.4)	
Female	97/212 (45.8)	14/30 (46.7)	83/182 (45.6)	
Occupation—No. (%)				≤0.001
Employee	57/212 (26.9)	2/30 (6.7)	55/182 (30.2)	0.007
Self-employed	8/212 (3.8)	0/30 (0)	8/182 (4.4)	0.382
Retired	96/212 (45.3)	24/30 (80)	72/182 (39.6)	<0.001
Unemployment	51/212 (24.1)	4/30 (13.3)	47/182 (25.8)	0.175
Medical staff—No. (%)	5/212 (2.4)	0/30 (0)	5/182 (2.7)	0.606
Disease status				
Mild	74/212 (34.9)	1/30 (3.3)	73/182 (40.1)	<0.001
Severe	117/212 (55.1)	11/30 (36.6)	106/182 (58.2)	0.030
Critical	22/212 (10.3)	18/30 (60)	4/182 (2.1)	<0.001
BMI-median—No. (%)				0.008
<25	150/212 (70.8)	20/29 (69)	130/166 (78.3)	0.270
25–30	36/212 (17)	4/29 (13.8)	32/166 (19.3)	0.606
>30	9/212 (4.2)	5/29 (17.2)	4/166 (2.4)	0.003
Temperature (°C)—No. (%)				0.003
≤37.00	95/212 (44.8)	5/30 (16.7)	90/182 (49.5)	0.001
37.01–38.00	62/212 (29.2)	12/30 (40)	50/182 (27.5)	0.162
38.01–39.00	45/212 (21.2)	9/30 (30)	36/182 (19.8)	0.205
≥39.01	10/212 (4.7)	4/30 (13.3)	6/182 (3.3)	0.038
Hypnotics—No. (%)				<0.001
Yes	68/212 (32.1)	2/30 (6.7)	66/153 (43.1)	<0.001
No	115/212 (54.2)	28/30 (93.3)	87/153 (56.9)	0.001
Smoking history—**N**o. (%)	30/212 (14.2)	8/30 (26.7)	22/182 (12.1)	0.034
Drinking—No. (%)	24/212 (11.3)	5/30 (16.7)	19/182 (10.4)	0.373
Signs and symptoms—No. (%)				
Fever	181/212 (85.4)	21/30 (70)	160/182 (87.9)	0.010
Cough	106/212 (50)	16/30 (53.3)	90/182 (49.5)	0.694
Fever and cough	177/212 (83.5)	16/30 (53.3)	161/182 (88.5)	<0.001
Chest distress	2/212 (0.9)	2/30 (6.7)	0/182 (0)	0.019
Nausea and vomiting	1/212 (0.5)	1/30 (3.3)	0/182 (0)	0.144
Dyspneic	10/212 (4.7)	1/30 (3.3)	9/182 (4.9)	1.000
Chronic medical illness/coexisting conditions—No. (%)				
Cirrhosis	4/212 (1.9)	2/30 (6.7)	2/182 (1.1)	0.105
Hypertension	76/212 (35.8)	14/30 (46.7)	62/182 (34.1)	0.182
Diabetes	38/212 (17.9)	12/30 (40)	26/182 (14.3)	0.001
Malignancy	3/212 (1.4)	2/30 (6.7)	1/182 (0.5)	0.055
Cerebrovascular disease	6/212 (2.8)	0/30 (0)	6/182 (3.3)	0.586
Chronic obstructive pulmonary disease	5/212 (2.4)	0/30 (0)	5/182 (2.7)	0.592
Chronic kidney disease	6/212 (2.8)	0/30 (0)	6/182 (3.3)	0.607
Chronic liver disease	2/212 (0.9)	0/30 (0)	2/182 (1.1)	1.000
Cardiovascular and cerebrovascular diseases	30/212 (14.2)	9/30 (30)	21/182 (11.5)	0.007
Digestive system disease	16/212 (7.5)	6/30 (20)	10/182 (5.5)	0.005
Endocrine system disease	12/212 (5.7)	1/30 (3.3)	11/182 (6)	0.698
Nervous system disease	6/212 (2.8)	2/30 (6.7)	4/182 (2.2)	0.200
Respiratory system disease	18/212 (8.5)	6/30 (20)	12/182 (6.6)	0.015

**Table 2:** Radiographic and laboratory findings of patients with COVID-19.

Radiologic and laboratory findings	All patients	Clinical outcomes		*p* value
		Septic shock	Nonseptic shock	
	212	30	182	
Radiologic findings				
Abnormalities on chest CT—No./total No. (%)				
Combination of patchy ground-glass opacity and pulmonary consolidation	39/212 (18.4)	1/30 (3.3)	38/182 (20.9)	0.019
Crazy paving sign	19/212 (9)	6/30 (20)	13/182 (7.1)	0.022
Bilateral pulmonary multiple consideration and intralobular interstitial thickening	7/212 (3.3)	6/30 (20)	1/182 (0.5)	<0.001
Laboratory findings				
White blood cell count, × 10^9^/L				≤0.001
<4	54/212 (25.5)	5/29 (17.2)	49/176 (27.8)	0.263
4–10	133/212 (62.7)	14/29 (48.3)	119/176 (67.6)	0.043
>10	18/212 (8.5)	10/29 (34.5)	8/176 (4.5)	<0.001
Neutrophil count, × 10^9^/L				0.005
<40	52/212 (24.5)	5/29 (17.2)	47/176 (26.7)	0.369
40–75	77/212 (36.3)	5/29 (17.2)	72/176 (40.9)	0.013
>75	76/212 (35.8)	19/29 (65.5)	57/176 (32.4)	0.001
Lymphocyte count, × 10^9^/L				0.066
<20	133/212 (62.7)	25/29 (86.2)	108/176 (61.4)	0.008
20–50	71/212 (33.5)	4/29 (13.8)	67/176 (38.1)	0.007
>50	1/212 (0.5)	0/29 (0)	1/176 (0.6)	1.000
Monocyte count, × 10^9^/L				0.635
<3	78/212 (36.8)	12/29 (41.4)	66/175 (37.7)	0.707
3–10	101/212 (47.6)	15/29 (51.7)	86/175 (49.1)	0.797
>10	25/212 (11.8)	2/29 (6.9)	23/175 (13.1)	0.411
Platelet count, × 10^9^/L				0.009
<100	12/212 (5.7)	5/29 (17.2)	7/175 (4)	0.018
100–300	152/212 (71.7)	22/29 (75.9)	130/175 (74.3)	0.857
>300	40/212 (18.9)	2/29 (6.9)	38/175 (21.7)	0.063
Activated partial thromboplastin time, s	28.8 (23–39.25)	34.55 (27.1–41.825)	27.7 (20.3–38.8)	<0.001
Creatine kinase—CMB, U/L	23.9 (21.65–25.1)	23.6 (21.2–24.7)	41.4 (24.2–45.6)	<0.001
Lactate, mmol/L	1.2 (1.2–1.3)	1.2 (1.2–1.3)	2.1 (1.8–2.1)	<0.001
Alanine aminotransferase, U/L				0.023
<7	36/212 (17)	0/29 (0)	36/177 (20.3)	0.006
7–40	125/212 (59)	20/29 (69)	105/177 (59.3)	0.324
>40	45/212 (21.2)	9/29 (31)	36/177 (20.3)	0.196
Aspartate aminotransferase, U/L				0.008
<13	40/212 (18.9)	1/29 (3.4)	39/178 (21.9)	0.024
13–35	101/212 (47.6)	12/29 (41.4)	89/178 (50)	0.389
>35	66/212 (31.1)	16/29 (55.2)	50/178 (28.1)	0.004
Blood urea nitrogen, mmol/L				0.040
<3	28/212 (13.2)	2/29 (6.9)	26/176 (14.8)	0.397
3–8	117/212 (55.2)	13/29 (44.8)	104/176 (59.1)	0.150
>8	60/212 (28.3)	14/29 (48.3)	46/176 (26.1)	0.015
Creatinine, *μ*mol/L				0.030
<88	170/212 (80.2)	19/29 (65.5)	151/177 (85.3)	0.009
88–144	31/212 (14.6)	8/29 (27.6)	23/177 (13)	0.042
>144	5/212 (2.4)	2/29 (6.9)	3/177 (1.7)	0.144
Glucose, mmol/L				<0.001
<3.9	35/212 (16.5)	0/26 (0)	35/176 (19.9)	0.006
3.9–6.1	84/212 (39.6)	5/26 (19.2)	79/176 (44.9)	0.020
>6.1	83/212 (39.2)	21/26 (80.8)	62/176 (35.2)	<0.001

^†^Data were missing for lactate dehydrogenase in 152 (71.6%).

**Table 3:** Treatments, complications, and clinical outcome.

	All patients	Clinical outcomes		*p* value
		Septic shock	Nonseptic shock	
	212	30	182	
Treatment				
Antiviral therapy—No. (%)				
Oseltamivir	143/212 (67.5)	23/30 (76.7)	120/182 (65.9)	0.245
Ganciclovir	144/212 (67.9)	21/30 (70)	123/182 (67.6)	0.793
Arbidol	153/212 (72.2)	17/30 (56.7)	136/182 (74.7)	0.041
Kaletra	23/212 (10.8)	11/30 (36.7)	12/182 (6.6)	≤0.001
Interferon	15/212 (7.1)	4/30 (13.3)	11/182 (6)	0.228
Antibiotic therapy—No. (%)				
Antibiotics	202/212 (95.3)	29/30 (96.7)	173/182 (95.1)	1.000
Use of corticosteroid/glucocorticoid therapy—No. (%)				
Corticosteroid/glucocorticoid	137/212 (64.6)	27/30 (90)	110/182 (60.4)	0.002
Continuous renal replacement therapy—No. (%)				
Oxygen support—No. (%)				
Nasal cannula	210/212 (99.1)	29/30 (96.7)	181/182 (99.5)	0.276
Noninvasive ventilation (NIV)	33/212 (15.6)	27/30 (90)	6/182 (3.3)	<0.001
Invasive ventilation (IV)	19/212 (9)	17/30 (56.7)	2/182 (1.1)	<0.001
Complication—No. (%)				
Acute cardiac injury	37/212 (17.5)	24/30 (80)	13/182 (7.1)	<0.001
Arrhythmia	21/212 (9.9)	18/30 (60)	3/182 (1.6)	<0.001
Acute respiratory distress syndrome	76/212 (35.8)	28/30 (93.3)	48/182 (26.4)	<0.001
Acute kidney injury	11/212 (5.2)	9/30 (30)	2/182 (1.1)	<0.001
Acute respiratory injury	76/212 (35.8)	29/30 (96.7)	47/182 (25.8)	<0.001
Secondary infection	9/212 (4.2)	8/30 (26.7)	1/182 (0.5)	<0.001

## Data Availability

Data are available on request from the authors.

## Supplementary Materials

Supplementary Materials Supplementary Figure S1 Clinical outcomes with hypnotics. Supplementary Figure S2. Illustrative chest computer tomography (CT) scans from one COVID-19 patients with septic shock. Supplementary Table S1. Radiographic and laboratory findings of patients with COVID-19. Supplementary Figure S3 Dynamic Profile of Laboratory test.
