# Supplementary material for: Association of Septic Shock with Mortality in Hospitalized COVID-19 Patients in Wuhan, China
Source: Adv Virol. 2022 Apr 23;2022:3178283. doi: 10.1155/2022/3178283 (PMC9056262; doi:10.1155/2022/3178283)
Supplement: Supplementary Materials — Supplementary Figure S1 Clinical outcomes with hypnotics. Supplementary Figure S2. Illustrative chest computer tomography (CT) scans from one COVID-19 patients with septic shock. Supplementary Table S1. Radiographic and laboratory findings of patients with COVID-19. Supplementary Figure S3 Dynamic Profile of Laboratory test. [file 3178283.f1.pdf]

Supplementary Appendix

Association of Septic Shock with Mortality in Hospitalized COVID-19 Patients in Wuhan, China

Medications

Dexzopiclone was administrated depending on the suggested dose, 1 mg/per day at slumber time. Antibiotics (moxifloxacin), corticosteroid/glucocorticoid according to their clinical presentation Antiviral medicine, ganciclovir, oseltamivir, Arbidol®, Kaletra® (lopinavir/ritonavir, 200 mg/500mg bid), interferon-α (aerosol inhalation, 2 mL bid). Dose and correct method were as counseled in the guidelines of COVID-19 diagnosis and treatment trial (5th edition), by the National Health Commission of the People's Republic of China.

Supplementary Figure S1

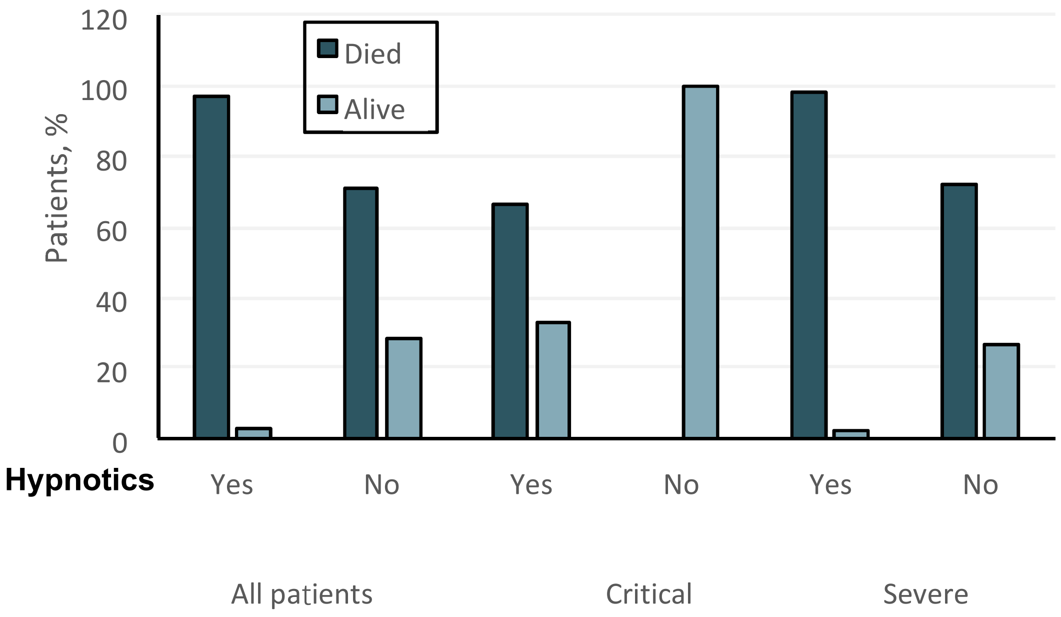

Supplementary Figure S1 Clinical outcomes with hypnotics

The survival rates of patients who took hypnotics shown in critical and severe severity were 66.7% and,98.2%. Of 100% ,27.3% of Non-hypnotics patients died in the critical and severe group.

Supplementary Figure S2

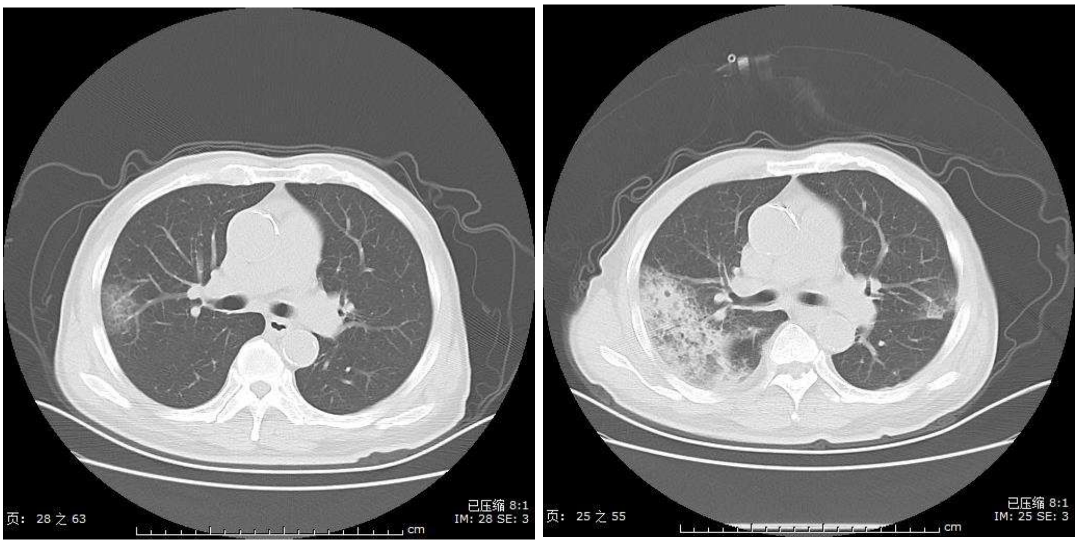

Figure S2. Illustrative chest computer tomography (CT) scans from one COVID-19 patients with septic shock. The picture on the left was collected when the patient was first admitted to the hospital, and on the right is five days later. The figure clearly shows that 1. Under the right lung pleura, see a patchy membrane glass shadow. 2. The lesions of both lungs increased more than before, and the lesions of the right lung progressed more than before.

Supplementary Table S1. Radiographic and laboratory findings of patients with COVID-19				
		**Clinical outcome**		**p Value**
**Radiologic and laboratory findings**	**All Patients**	**Septic Shock**	**Non-Septic Shock**	
	212	**30**	**182**	
**Radiologic findings**				
**Abnormalities on chest CT – No./total No. (%)**				
No GGO	2/212(0.9)	0/30(0)	2/182(1.1)	1.000
Local GGO	8/212(3.8)	1/30(3.3)	7/182(3.8)	1.000
Bilateral GGO	111/212(52.4)	11/30(36.7)	100/182(54.9)	0.063
Combination of patchy ground-glass opacity and pulmonary consolidation	39/212(18.4)	1/30(3.3)	38/182(20.9)	0.019
Crazy paving sign	19/212(9)	6/30(20)	13/182(7.1)	0.022
Diffuse patchy ground glass and air bronchogram	20/212(9.4)	3/30(10)	17/182(9.3)	1.000
Bilateral pulmonary multiple consideration and intralobular interstitial thickening	7/212(3.3)	6/30(20)	1/182(0.5)	<0.001
**Laboratory findings**				
PH*				0.377
<7.35	1/212(0.5)	1/11(9.1)	0/26(0)	0.308
7.35-7.45	11/212(5.2)	3/11(27.3)	8/26(30.8)	1.000
>7.45	25/212(11.8)	7/11(63.6)	18/26(69.2)	1.000
PaO2, mmHg *				0.505
<80	21/212(9.9)	6/11(54.5)	15/26(57.7)	1.000
80-100	6/212(2.8)	3/11(27.3)	3/26(11.5)	0.322
>100	10/212(4.7)	2/11(18.2)	8/26(30.8)	0.667
PaCO2, mmHg *				0.080
<35	22/212(10.4)	4/11(36.4)	18/26(69.2)	0.077
35-45	15/212(7.1)	7/11(63.6)	8/26(30.8)	0.094
PaO2/Fio2*				0.079
<400	31/212(14.6)	9/11(81.8)	22/25(88)	1.000

400-500	2/212(0.9)	2/11(18.2)	0/25(0)	0.089
>500	3/212(1.4)	0/11(0)	3/25(12)	0.545
**White blood cell count, $\times 10^9$/L**				0.000
<4	54/212(25.5)	5/29(17.2)	49/176(27.8)	0.263
4-10	133/212(62.7)	14/29(48.3)	119/176(67.6)	0.043
>10	18/212(8.5)	10/29(34.5)	8/176(4.5)	<0.001
**Neutrophil count, $\times 10^9$/L**				0.005
<40	52/212(24.5)	5/29(17.2)	47/176(26.7)	0.369
40-75	77/212(36.3)	5/29(17.2)	72/176(40.9)	0.013
>75	76/212(35.8)	19/29(65.5)	57/176(32.4)	0.001
**Lymphocyte count, $\times 10^9$/L**				0.066
<20	133/212(62.7)	25/29(86.2)	108/176(61.4)	0.008
20-50	71/212(33.5)	4/29(13.8)	67/176(38.1)	0.007
>50	1/212(0.5)	0/29(0)	1/176(0.6)	1.000
**Monocyte count, $\times 10^9$/L**				0.635
<3	78/212(36.8)	12/29(41.4)	66/175(37.7)	0.707
3-10	101/212(47.6)	15/29(51.7)	86/175(49.1)	0.797
>10	25/212(11.8)	2/29(6.9)	23/175(13.1)	0.411
**Platelet count, $\times 10^9$/L**				0.009
<100	12/212(5.7)	5/29(17.2)	7/175(4)	0.018
100-300	152/212(71.7)	22/29(75.9)	130/175(74.3)	0.857
>300	40/212(18.9)	2/29(6.9)	38/175(21.7)	0.063
**C-reactive protein level mg/liter – No./total No. (%)**				0.130
≤3	15/212(7.1)	0/30(0)	15/173(8.7)	0.143
>3	188/212(88.7)	30/30(100)	158/173(91.3)	0.133
**SAA mg/liter – No./total No. (%)**				0.078
≤10	19/212(9)	0/27(0)	19/169(11.2)	0.079

>10	177/212(83.5)	27/27(100)	150/169(88.8)	0.077
**Prothrombin time, s**				0.411
<9	15/212(7.1)	1/30(3.3)	14/161(8.7)	0.484
>14	30/212(14.2)	6/30(20)	24/161(14.9)	0.482
**Activated partial thromboplastin time, s**	28.8(23-39.25)	34.5(27.1-41.8)	27.7(20.3-38.8)	<0.001
**Creatine kinase–CMB, U/L**	23.9(21.65-25.1)	23.6(21.2-24.7)	41.4(24.2-45.6)	<0.001
**Lactate, mmol/L**	1.2(1.2-1.3)	1.2(1.2-1.3)	2.1(1.8-2.1)	<0.001
**D-dimer ≥ 0.5 mg/liter – No./total No. (%)**				0.907
≤0.5	92/212(43.4)	13/27(48.1)	79/162(48.8)	0.953
>0.5	96/212(45.3)	13/27(48.1)	83/162(51.2)	0.766
**Hypersensitive troponin I, pg/mL**				0.906
≤0.04	111/212(52.4)	18/28(64.3)	93/142(65.5)	0.902
>0.04	58/212(27.4)	9/28(32.1)	49/142(34.5)	0.809
**Lactate dehydrogenase, U/L †**				0.136
<120	37/212(17.5)	1/4(25)	36/52(69.2)	0.113
120 - 250	6/212(2.8)	1/4(25)	5/52(9.6)	0.372
>250	13/212(6.1)	2/4(50)	11/52(21.2)	0.237
**Alanine aminotransferase, U/L**				0.023
<7	36/212(17)	0/29(0)	36/177(20.3)	0.006
7-40	125/212(59)	20/29(69)	105/177(59.3)	0.324
>40	45/212(21.2)	9/29(31)	36/177(20.3)	0.196
**Aspartate aminotransferase, U/L**				0.008
<13	40/212(18.9)	1/29(3.4)	39/178(21.9)	0.024
13-35	101/212(47.6)	12/29(41.4)	89/178(50)	0.389
>35	66/212(31.1)	16/29(55.2)	50/178(28.1)	0.004
**Total bilirubin, mmol/L**				0.773
<5	13/212(6.1)	1/29(3.4)	12/178(6.7)	0.689

5-22	158/212(74.5)	22/29(75.9)	136/178(76.4)	0.949
>22	36/212(17)	6/29(20.7)	30/178(16.9)	0.613
**Blood urea nitrogen, mmol/L**				0.040
<3	28/212(13.2)	2/29(6.9)	26/176(14.8)	0.397
3-8	117/212(55.2)	13/29(44.8)	104/176(59.1)	0.150
>8	60/212(28.3)	14/29(48.3)	46/176(26.1)	0.015
**Creatinine, µmol/L**				0.030
<88	170/212(80.2)	19/29(65.5)	151/177(85.3)	0.009
88-144	31/212(14.6)	8/29(27.6)	23/177(13)	0.042
>144	5/212(2.4)	2/29(6.9)	3/177(1.7)	0.144
**Procalcitonin, ng/mL**				0.645
≤0.1	125/212(59)	17/25(68)	108/149(72.5)	0.645
>0.1	49/212(23.1)	8/25(32)	41/149(27.5)	0.645
**Glucose, mmol/L**				<0.001
<3.9	35/212(16.5)	0/26(0)	35/176(19.9)	0.006
3.9-6.1	84/212(39.6)	5/26(19.2)	79/176(44.9)	0.020
>6.1	83/212(39.2)	21/26(80.8)	62/176(35.2)	<0.001
**Potassium, mmol/L**				0.026
<3.5	54/212(25.5)	4/25(16)	50/171(29.2)	0.239
3.5-5.5	141/212(66.5)	20/25(80)	121/171(70.8)	0.368
>5.5	1/212(0.5)	1/25(4)	0/171(0)	0.126

*Data were missing for PH in 175(82.5%), for PaO2 in 175(82.5%), for PaCO2 in 175(82.5%), for PaO2:FiO2 in 176(83%)

†Data were missing for lactate dehydrogenase in 152(71.6%)

Supplementary Figure S3

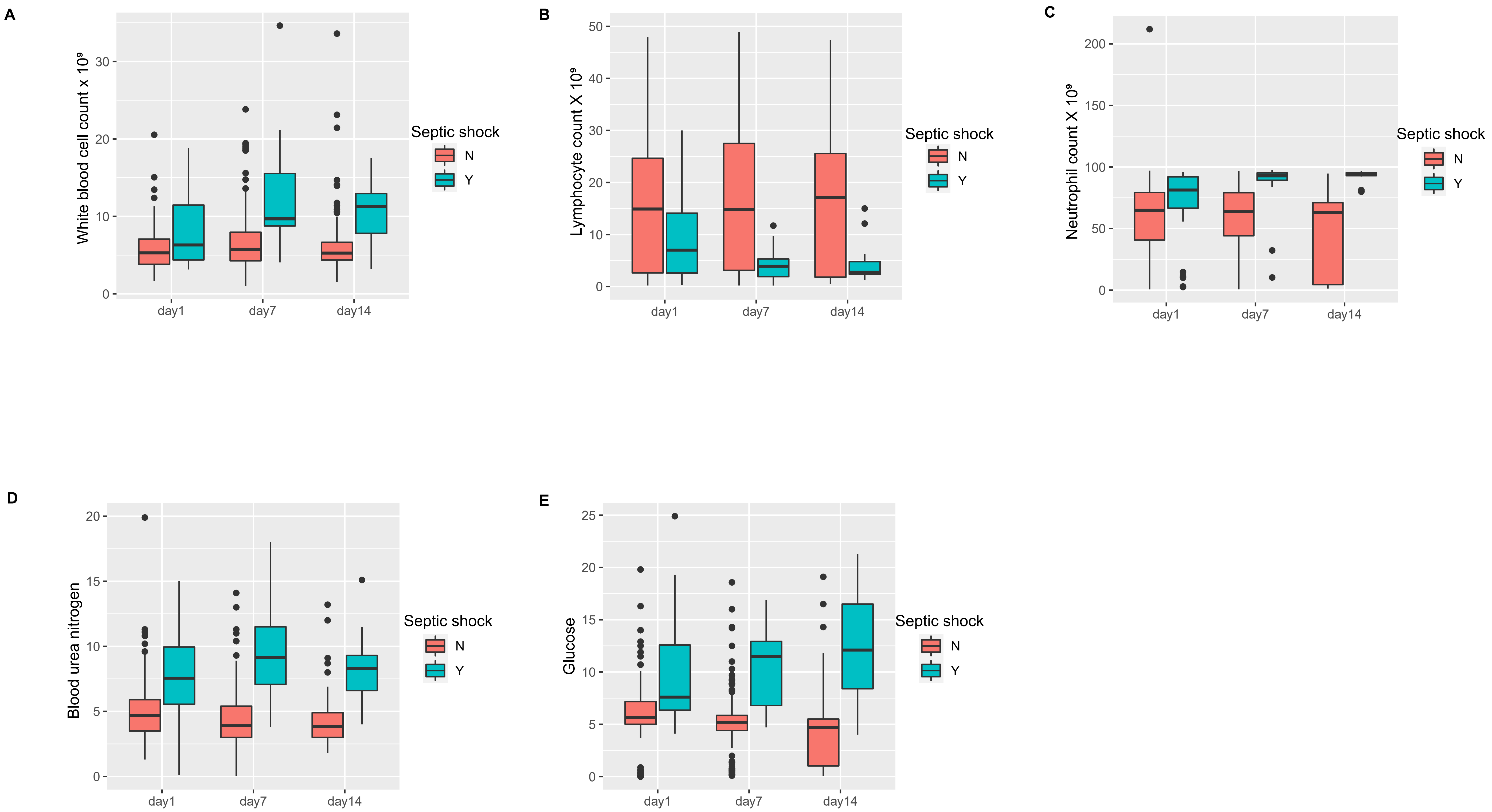

Supplementary Figure S3 Dynamic Profile of Laboratory test.
Fourteen days dynamic laboratory test results of COVID-19 patients with septic shock after hospitalization. (A) White blood cell count, (C) Neutrophil count, (E) Blood urea nitrogen, and (F) Glucose was significantly increased in the septic shock group. (B) Lymphocyte count was significantly decreased.
